# Supplementary material for: Evaluating acceptability of the Inpatient Mental Health Pharmaceutical Assessment and Care Tool (IMPACT): A multi-site study in the United Kingdom
Source: PLoS One. 2026 Feb 6;21(2):e0341776. doi: 10.1371/journal.pone.0341776 (PMC12880654; doi:10.1371/journal.pone.0341776)
Supplement: S1 File — (DOCX) [file pone.0341776.s001.docx]

**Supplementary File 1**

**Title:** Evaluating acceptability of the Inpatient Mental Health Pharmaceutical Assessment and Care Tool (IMPACT): a multi-site study in the United Kingdom

**Journal:** PLOS One

**Authors:** Fatima Q. Alshaikhmubarak^1^, Richard N. Keers^1,2,3^, Petra Brown^1,3^, Penny J. Lewis^1,2,4^

1. Division of Pharmacy and Optometry, The University of Manchester, Manchester, UK

2. NIHR Greater Manchester Patient Safety Research Collaboration, Manchester, UK

3. Optimising Outcomes with Medicines (OptiMed) Research Unit, Pennine Care NHS Foundation Trust, Manchester, UK.

4. Manchester University NHS Foundation Trust, Manchester, UK

**IMPACT tool version 1**

- Note that the appropriate review frequency will vary between and within organisations due to differences in staffing levels, workload, service capacity, and day-to-day operational pressures, which explains the overlap between the proposed ranges.
