# Supplementary material for: Evaluating acceptability of the Inpatient Mental Health Pharmaceutical Assessment and Care Tool (IMPACT): A multi-site study in the United Kingdom
Source: PLoS One. 2026 Feb 6;21(2):e0341776. doi: 10.1371/journal.pone.0341776 (PMC12880654; doi:10.1371/journal.pone.0341776)
Supplement: S2 File — (DOCX) [file pone.0341776.s002.docx]

**Supplementary File 2**

**Title:** Evaluating acceptability of the Inpatient Mental Health Pharmaceutical Assessment and Care Tool (IMPACT): a multi-site study in the United Kingdom

**Journal:** PLOS One

**Authors:** Fatima Q. Alshaikhmubarak^1^, Richard N. Keers^1,2,3^, Petra Brown^1,3^, Penny J. Lewis^1,2,4^

1. Division of Pharmacy and Optometry, The University of Manchester, Manchester, UK

2. NIHR Greater Manchester Patient Safety Research Collaboration, Manchester, UK

3. Optimising Outcomes with Medicines (OptiMed) Research Unit, Pennine Care NHS Foundation Trust, Manchester, UK.

4. Manchester University NHS Foundation Trust, Manchester, UK

**Table 1S** Detailed description of participating NHS organisations.

|  | NHS Borders | LSCFT | OHFT | PCFT | SWYT |
| --- | --- | --- | --- | --- | --- |
| Services | Inpatient: older adult, acute adult, and rehabilitation.  Outpatient: as above plus CAMHS | Acute adult, older adult, early intervention, rehabilitation, urgent care services, CAMHS, eating disorders, forensics, perinatal mental health, learning disability services. | Adult mental health, older adult, CAMHS, forensics, learning disability, rehabilitation. | Rehabilitation, CAMHS, later life, forensics, substance misuse, learning disabilities. | In and outpatient acute mental health, older people’s mental health, learning disability, forensics, CAMHS outpatients |
| Number of inpatient wards | 4 | 50 | 25 | 33+ | 35 |
| Wards distribution and geographical locations | 4 wards across 2 sites | 50 wards across 23 sites | 25 wards across 7 sites | 33+ inpatient wards across 7 sites | 35 wards across 5 hospital sites and three off site rehab units |
| Pharmacy team size | 2 Pharmacists  1 Pharmacy Technician | 57 Pharmacists (47.23WTE)  1 Trainee pharmacist (1WTE)  34 Pharmacy technicians (31.83 WTE)  2 Pre-Registration Trainee Pharmacy Technicians (2WTE)  3 Pharmacy Assistants (3 WTE)  Staff are based either on an inpatient site or at a community base. There is a requirement for some travel, particularly when covering leave or a team is split across multiple bases. These numbers are for current staff on post (excluding vacancies). | 18 Clinical mental health specialist pharmacists *  10 medicines management technicians based around our mental health wards  * Including those who work in outpatient teams and MI but not including those in community services, meds safety or chief pharmacist | 36 staff across community and in-patient services.  23 Pharmacists  2 Nurses  10 Pharmacy technicians  1 Administrator | 18 Pharmacists  18 Pharmacy technicians |
| Number of inpatient beds | 45 beds | 709 beds | 407 mental health beds | 486 | 528 |
| Inpatient pharmacists’ typical duties  Medication Reviews  Medication reconciliation  Clinical Advice  Discharge Planning  Monitoring and Surveillance  Patient Counselling  Drug Information Services  Medication related audits  Medication prescribing | All the duties listed. | All the duties listed.  Additional duties:   - Attendance at MDT meetings and CPA reviews - Audits - Education and Training - Clinical Supervision - Drug Histories - Policy development | All the duties listed. | All the duties listed. | All the duties listed. |
| Inpatient pharmacy technicians’ typical duties  Medication dispensing  Accuracy checking  Medication Reviews  Medication reconciliation  Clinical Advice  Discharge Planning  Monitoring and Surveillance  Patient Counselling  Drug Information Services  Medication related audits | All the duties listed. | All the duties listed (dispensing for leave and discharge only).  Additional duties:   - Ordering Medication - Drug histories - Incident reporting - Procedural reviews - Managing self-administration programmes | All the duties listed. | All the duties listed. | All the duties listed except:   - Medication Reviews - Drug Information Services   Additional duties:   - Service improvement projects |
| EPMA | Not implemented | Implemented on all inpatient mental health wards. CLEO being rolled out to all community mental health teams | Implemented on all mental health wards (inpatient only) | One ward only | Fully implemented across all inpatient wards |
| Dispensary | Dispensing done via acute hospital dispensary onsite (i.e. NOT a dedicated mental health dispensary) | Mix- service level agreements in most localities, stocks ordered in-house on one site, dispensary on another site to manage all but clozapine patients | In house dispensary | Service level agreements only | In house dispensary, plus service level agreement with a community pharmacy for one site, and service level agreement with local general hospital for another site. |
| LSCFT: Lancashire and South Cumbria NHS Foundation Trust, OHFT: Oxford Health NHS Foundation Trust PCFT: Pennine Care NHS Foundation Trust, SWYT: South West Yorkshire Partnership NHS Foundation Trust, CAMHS: Child and Adolescent Mental Health Services, PICU: Psychiatric Intensive Care Units. | | | | | |
