# Supplementary material for: Evaluating acceptability of the Inpatient Mental Health Pharmaceutical Assessment and Care Tool (IMPACT): A multi-site study in the United Kingdom
Source: PLoS One. 2026 Feb 6;21(2):e0341776. doi: 10.1371/journal.pone.0341776 (PMC12880654; doi:10.1371/journal.pone.0341776)
Supplement: S3 File — (DOCX) [file pone.0341776.s003.docx]

**Supplementary File 3**

**Title:** Evaluating acceptability of the Inpatient Mental Health Pharmaceutical Assessment and Care Tool (IMPACT): a multi-site study in the United Kingdom

**Journal:** PLOS One

**Authors:** Fatima Q. Alshaikhmubarak^1^, Richard N. Keers^1,2,3^, Petra Brown^1,3^, Penny J. Lewis^1,2,4^

1. Division of Pharmacy and Optometry, The University of Manchester, Manchester, UK

2. NIHR Greater Manchester Patient Safety Research Collaboration, Manchester, UK

3. Optimising Outcomes with Medicines (OptiMed) Research Unit, Pennine Care NHS Foundation Trust, Manchester, UK.

4. Manchester University NHS Foundation Trust, Manchester, UK

**Focus group guide**

**Introduction:**

1. Introduce yourself.

2. Introduce the aim of the study:

**Aim:** This study aims to explore the prospective acceptability of the patient prioritisation model by mental health pharmacy team members.

3. Introduce the objectives of the focus group:

A focus group is a conversation that focuses on specific questions. In the study, we are interested in your views and opinions on the patient prioritisation model. I will guide the conversation by asking questions that each of you can respond to and discuss as a group.

4. Seek informed consent.

5. Ground rules:

- The focus group will be recorded, kindly speak clearly and make sure only one person speaks at a time so we can transcribe accurately.
- There are no right or wrong answers, only differing points of view.
- You don't need to agree with others, but you must listen respectfully as others share their views.
- If you have a pressing comment, please write it down so you do not forget it when it’s your turn to speak.
- Kindly mute your phones if possible, and if you must respond to a call, please do so as quietly as possible and rejoin us as quickly as you can.
- My role as moderator will be to guide the discussion while you talk to each other.
- Keep in mind that we're just as interested in constructive comments as positive comments, and at times the more constructive comments are the most helpful.

6. Ask interviewees whether they would like to ask anything before the start of the interview.

7. Signal the start of the interview and start recording.

**Start recording**

**Background questions:**

1. Let’s start by introducing ourselves one at a time. Could you please tell us your name, your role, and which group of patients do usually work with?

**Affective attitude:**

1. Could you tell me how comfortable you felt using the patient prioritisation model? **Prompt:** How naturally did it come to you, and did you find it to be a pleasant exercise??
2. Could you tell me what made you feel this way?

**Burden:**

1. How easy was it to use the model? Prompt: **Why?**
2. Do you think it would require some/more training/support? **Prompt:** What sort of training/support?
3. What barriers prevented you from using the model?

**Ethicality:**

1. Do you think the use of this model is fair to patients? **Prompt:** please explain your answers

**Perceived effectiveness:**

1. Do you think the use of this model will improve patient outcomes? **Prompt:** In what way?
2. Do you think the use of this model will improve pharmacy service delivery? **Prompt:** How?
3. Do you feel the model adequately represents medicines optimisation and safety issues affecting patients? **Prompt:** Do you think some elements of the model could be modified/removed/added to better reflect the ratings of patients?

**Self-efficacy:**

1. How confident did you feel using this model? **Prompt:** Please describe why you feel this way and whether this changed over time

**Opportunity cost:**

1. How much time did it take you to complete the assessment? **Prompt:** What influenced how long the assessment took to complete? Could the process be faster, and if so what could speed up the process?
2. Do you think the time spent on completing the model was well spent considering its benefits?

**General acceptability:**

1. How acceptable was the model in general? **Prompt:** what’s your overall opinion? Are you satisfied with the model and willing to use it?
2. Could you think of anything that may improve how acceptable it is to use?
3. Could you share your thoughts on how this model could be implemented and utilised in the future, considering potential benefits and challenges?

**Conclusion**
1. Summarise the discussion.
2. Ask if anything has been missed.
3. Thank everyone and stop recording.

**Thank you very much for your time, this was very insightful, I will stop recording now.**

**Explain what happens next:** The interview will be transcribed and analysed to better understand and improve the acceptability of the model.
