# Supplementary material for: Evaluating acceptability of the Inpatient Mental Health Pharmaceutical Assessment and Care Tool (IMPACT): A multi-site study in the United Kingdom
Source: PLoS One. 2026 Feb 6;21(2):e0341776. doi: 10.1371/journal.pone.0341776 (PMC12880654; doi:10.1371/journal.pone.0341776)

**Supplementary File 4**

**Title:** Evaluating acceptability of the Inpatient Mental Health Pharmaceutical Assessment and Care Tool (IMPACT): a multi-site study in the United Kingdom

**Journal:** PLOS One

**Authors:** Fatima Q. Alshaikhmubarak^1^, Richard N. Keers^1,2,3^, Petra Brown^1,3^, Penny J. Lewis^1,2,4^

1. Division of Pharmacy and Optometry, The University of Manchester, Manchester, UK

2. NIHR Greater Manchester Patient Safety Research Collaboration, Manchester, UK

3. Optimising Outcomes with Medicines (OptiMed) Research Unit, Pennine Care NHS Foundation Trust, Manchester, UK.

4. Manchester University NHS Foundation Trust, Manchester, UK

**Changes to the training and IMPACT tool version 1**

**Changes to IMPACT tool training version 1**

- Added more instructions/ training to the tool:

1. The example of using the tool was expanded by including simulated patient information to apply the tool for.
2. The following notes and considerations were added:

- The tool is intended to be used on admission (during or after medication reconciliation) and then reviewed every time the patient is seen.
- The tool should be used flexibly (e.g. blood tests may not be available but it is there for you to complete it when you can)
- The frequency of review is just for guidance, and the tool might help you spend more time with red patients rather than reviewing all patients quickly.
- This tool was primarily developed for use in adult wards so some criteria may not be applicable, future work may explore speciality specific tools.
- It expected that professional judgement is used when necessary and that ‘other’ allows for the professional to escalate or deescalate patients if required- the tool cannot cover every patient presentation and risk.
- This evaluation is key in improving the tool and making sure it is practical to use.

**IMPACT tool version 2**

- Changes are highlighted in yellow.
- Combined risk indicators are highlighted in blue.


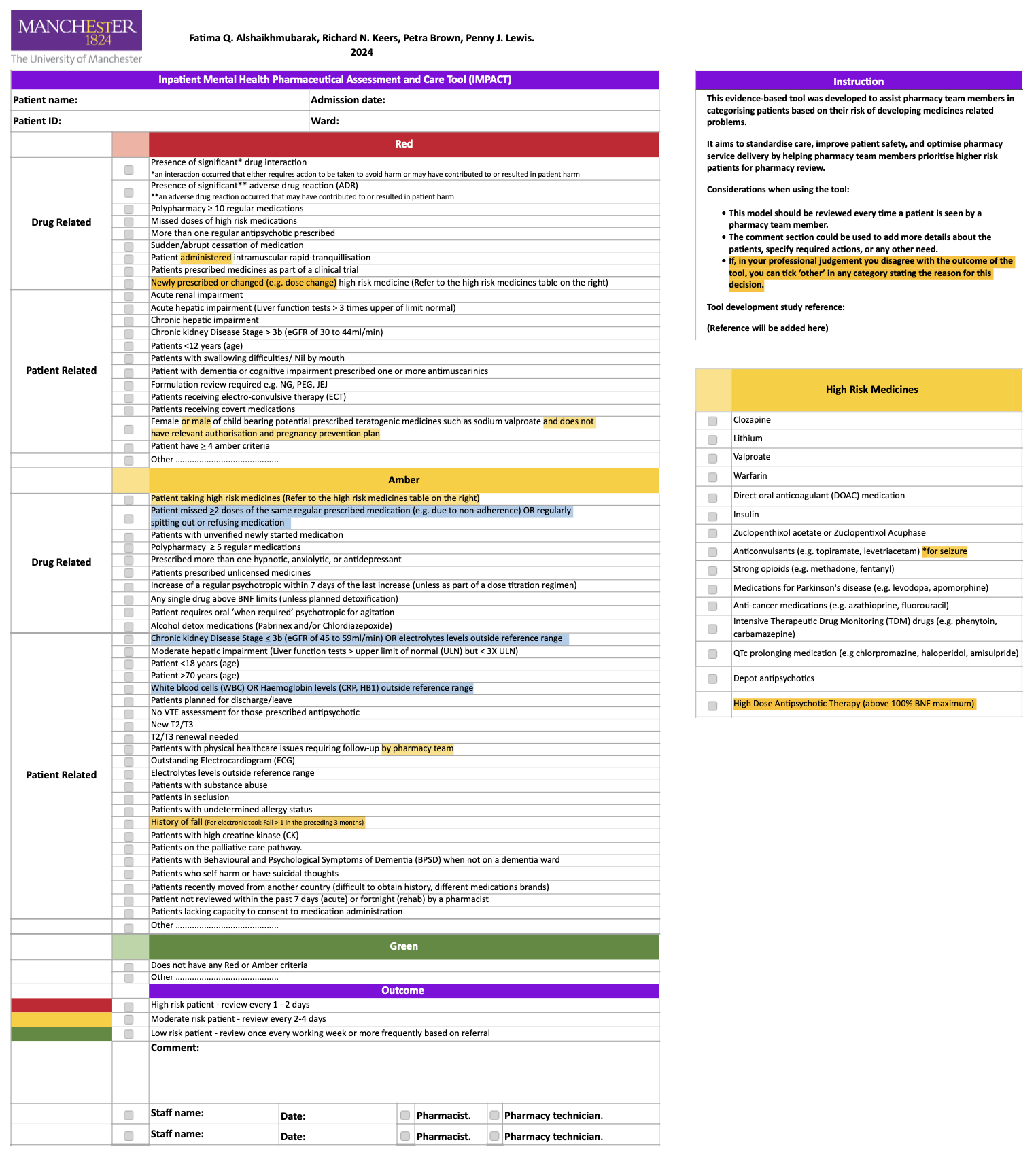


**Training session material version 2**


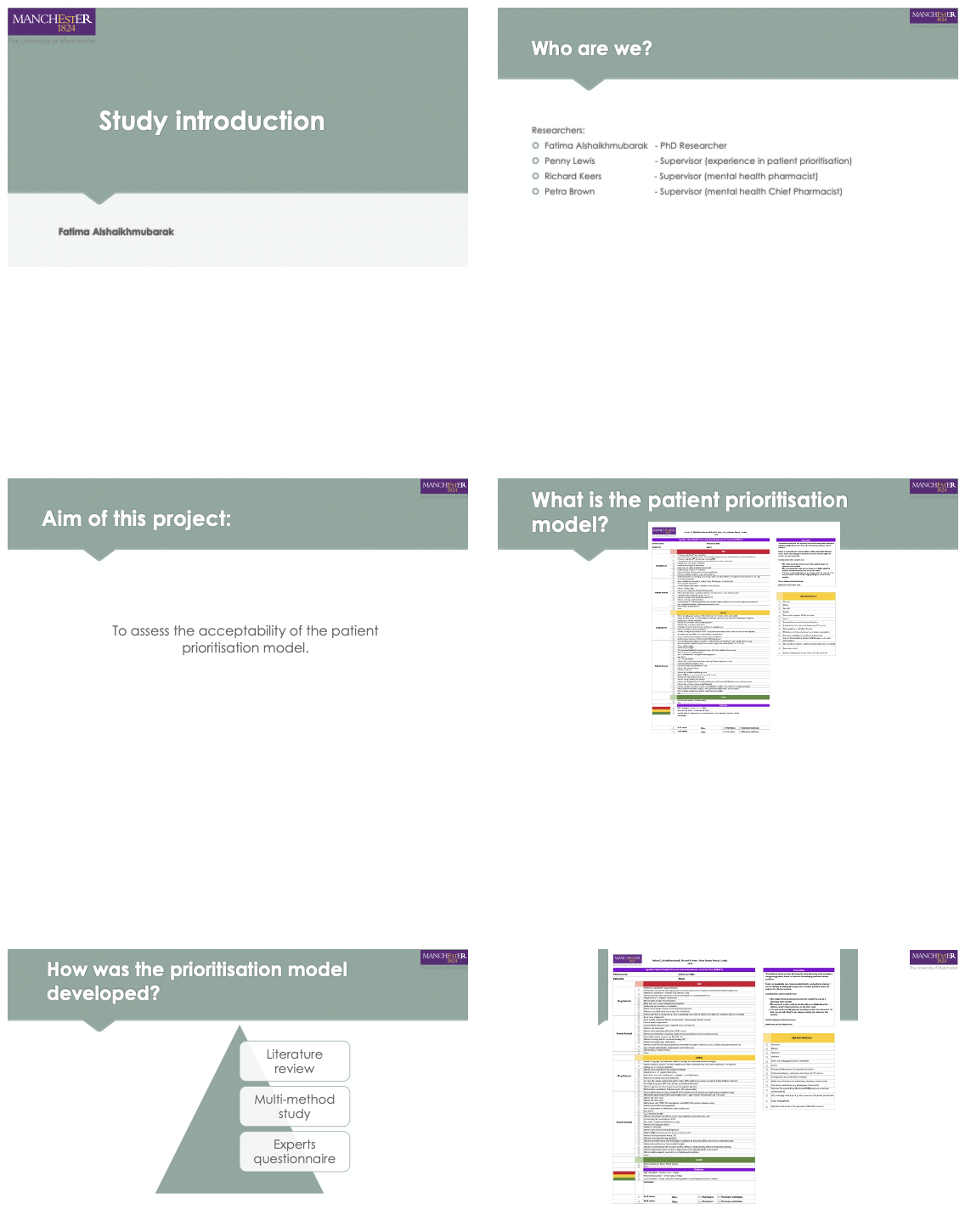


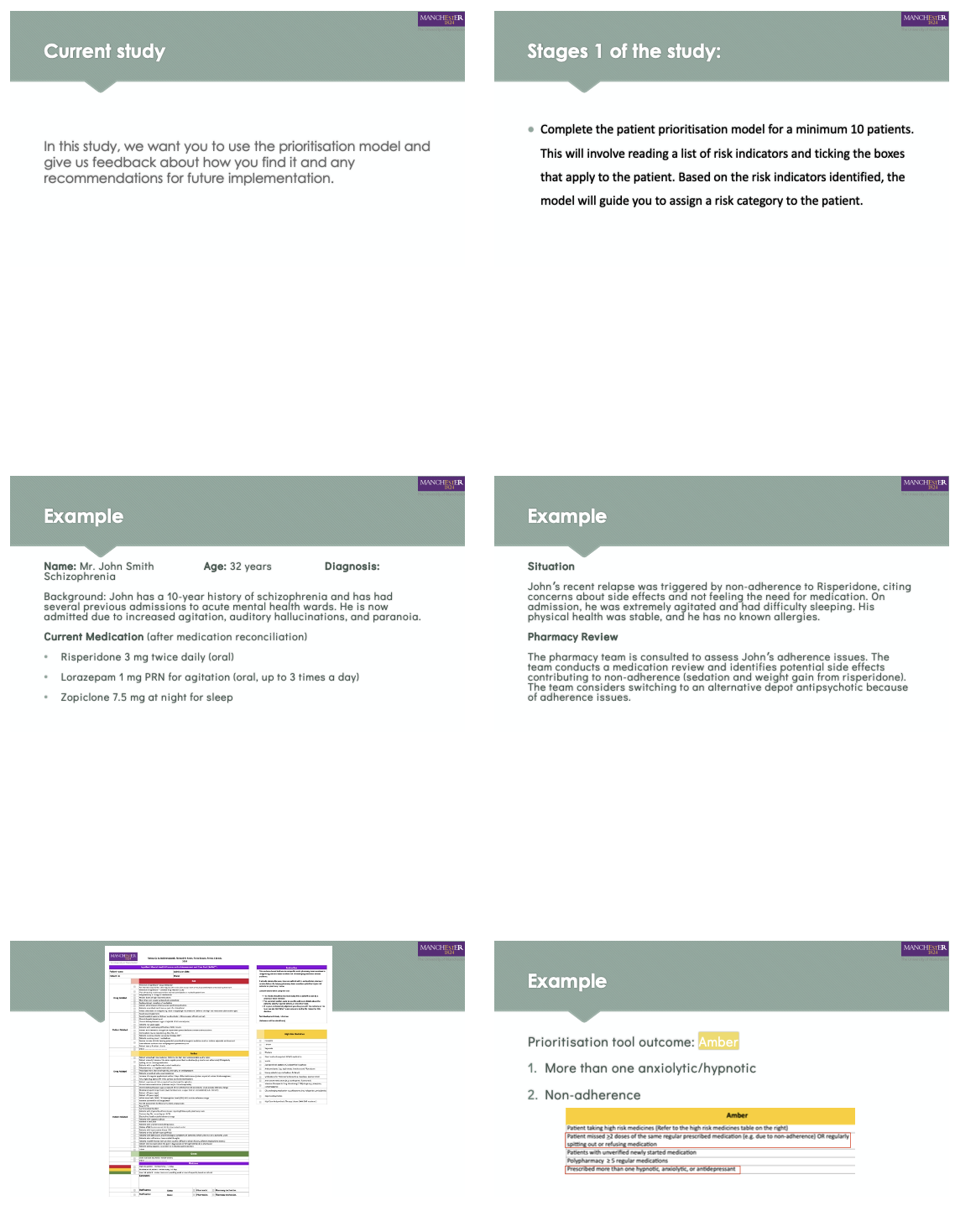


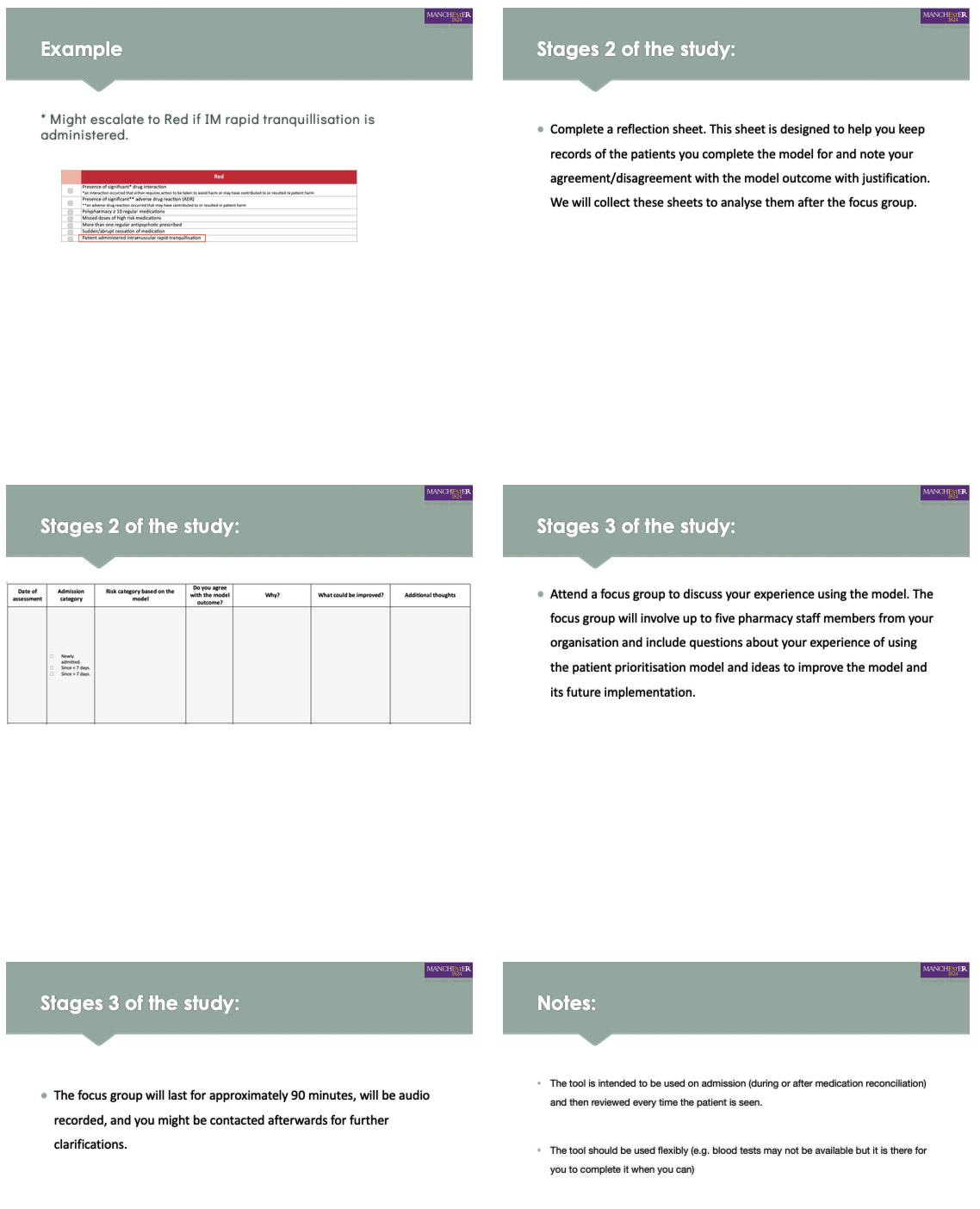


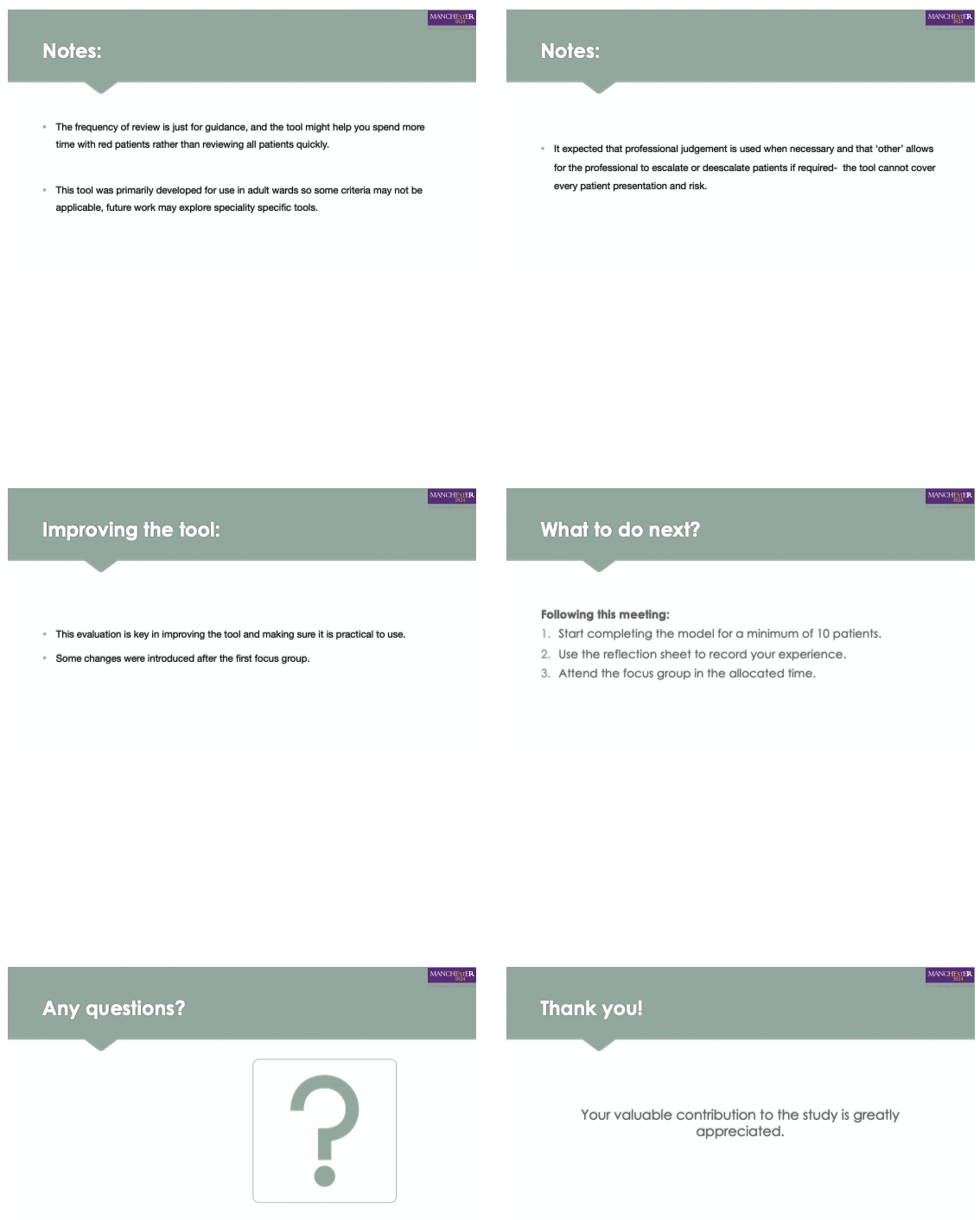

Supplement: S4 File — (DOCX) [file pone.0341776.s004.docx]
