# Supplementary material for: Evaluating acceptability of the Inpatient Mental Health Pharmaceutical Assessment and Care Tool (IMPACT): A multi-site study in the United Kingdom
Source: PLoS One. 2026 Feb 6;21(2):e0341776. doi: 10.1371/journal.pone.0341776 (PMC12880654; doi:10.1371/journal.pone.0341776)
Supplement: S5 File — (DOCX) [file pone.0341776.s005.docx]

**Supplementary File 5**

**Title:** Evaluating acceptability of the Inpatient Mental Health Pharmaceutical Assessment and Care Tool (IMPACT): a multi-site study in the United Kingdom

**Journal:** PLOS One

**Authors:** Fatima Q. Alshaikhmubarak^1^, Richard N. Keers^1,2,3^, Petra Brown^1,3^, Penny J. Lewis^1,2,4^

1. Division of Pharmacy and Optometry, The University of Manchester, Manchester, UK

2. NIHR Greater Manchester Patient Safety Research Collaboration, Manchester, UK

3. Optimising Outcomes with Medicines (OptiMed) Research Unit, Pennine Care NHS Foundation Trust, Manchester, UK.

4. Manchester University NHS Foundation Trust, Manchester, UK

**The Inpatient Mental Health Pharmaceutical Assessment and Care Tool (IMPACT) - User Manual**

Table of Contents

[1. Introduction 2](#_Toc194513641)

[2. Benefits of using patient prioritisation tools 2](#_Toc194513642)

[3. Getting Started with the IMPACT tool 3](#_Toc194513643)

[4. Using the IMPACT tool 3](#_Toc194513644)

[5. Implementation of the IMPACT tool 6](#_Toc194513645)

[6. Documentation of IMPACT tool use 7](#_Toc194513646)

[7. Feedback and Improvement 7](#_Toc194513647)

[8. Training 7](#_Toc194513648)

[9. Adaptation of the IMPACT tool for Different Contexts 7](#_Toc194513649)

[10. Support mechanisms 9](#_Toc194513650)

[11. Frequently asked questions 9](#_Toc194513651)

# **1. Introduction**

- **Welcome:**
  - Welcome to the IMPACT tool manual. This evidence-based tool was developed to assist pharmacy team members in categorising patients for pharmacy review based on their risk of developing medicines related problems.
  - The IMPACT tool aims to standardise care, improve patient safety, and optimise pharmacy service delivery by helping pharmacy team members prioritise higher risk patients for pharmacy review.
- **Purpose:**
  - This manual provides instructions on how to use the IMPACT tool effectively, ensuring accurate and consistent patient prioritisation.
- **Safety:**
  - The IMPACT tool was developed to support clinical judgment; it is not a replacement for professional assessment. Always use clinical judgement.
- **Development:**
  - The IMPACT tool was developed through several studies, outlined below. It is worth noting that a group of stakeholders (two pharmacists, two patient representatives with lived experience in mental health inpatient wards, and one pharmacy technician) assisted in developing the tool (during steps 4&5).
  1. Initially, a systematic review of the literature was conducted to identify what indicators make mental health inpatients at higher risk of medication harm (Alshaikhmubarak et al., 2023).
  2. This was followed by interviews with pharmacists working for mental health organisations across the UK to explore how they prioritise care for their inpatients, which provided insights into the tool development and implementation.
  3. Identified patient prioritisation tools/processes from step 2 above were analysed to identify more risk indicators.
  4. Questionnaires with experts in mental health care and medicines were used to obtain agreement on what risk indicators (identified from step 1,2, and 3) should be included in the tool and how to categorise them into risk groups.
  5. Questionnaires with pharmacy team experts were used to obtain agreement on the design of the tool and practical issues regarding how to use the tool (e.g. training, frequency of review).

# **2. Benefits of using patient prioritisation tools**

Outside of mental health care, in UK acute hospital care, pharmaceutical patient prioritisation tools were reported to:

- Improve patient care and ensure that the right patients were seen at the right time.
- Improve pharmacy service delivery and workload prioritisation.

In mental health inpatient settings, our research exploring the use of local prioritisation approaches by pharmacy teams has identified that:

- Those we spoke to believed their own local prioritisation tool/process successfully achieved their aims which were improving patient safety, optimising pharmacy service delivery, and standardising care.
- One pharmacist reported receiving fewer patient safety medication incident reports after implementing their own local prioritisation tool.
- Local prioritisation tools/processes were found to be valuable for managers in allocating resources effectively and providing evidence to secure funding and support expansion of pharmacy staff.

While the identified prioritisation approaches were found to be very useful, they had limitations, mainly lack of strong evidence. Therefore, the IMPACT tool was developed through learning from existing approaches and evidence as described in section 1. Evaluation of the IMPACT tool identified positive views from participating pharmacy staff and identified additional benefits to using it such as:

- Facilitating handover
- Supporting benchmarking
- Enhancing accountability
- Supporting staffing allocation.

# **3. Getting Started with the IMPACT tool**

The IMPACT tool can be printed and used in paper format or incorporated into an Excel sheet, offering flexibility in your workflow.

- **Printed Format:**
  - Print the checklist from the provided file.
  - Agree on a process for documentation and handover with your team.
- **Excel Format:**
  - Convert the pdf file to an MS Excel format either automatically or manually.
  - Save the file in a shared folder or somewhere accessible by all team members.
  - Agree on how to document and handover the tool for each inpatient.

# **4. Using the IMPACT tool**

- **What is the purpose of the IMPACT tool?**
  - The IMPACT tool was developed to assist pharmacy team members in categorising patients for pharmacy review based on their risk of developing medicines related problems.
  - The main purpose of the tool is to categorise patients into risk groups to help pharmacy team members pay more attention to higher risk patients, consequently improving patients’ outcomes. (This is based on the understanding that patients may require different levels of attention from pharmacy teams to achieve the same outcomes).
- **When to use the IMPACT tool?**
  - This tool should be completed for all inpatients on admission, either during or after medication reconciliation. It should then be reviewed and updated every time a patient is seen by a pharmacy team member.
- **Who should use the IMPACT tool?**
  - When developing the IMPACT tool, experts agreed that it should be used by both pharmacists and pharmacy technicians alike. However, it should be locally agreed whether pharmacy technicians should use the tool and to provide the appropriate training to support them in using the tool effectively.
- **IMPACT tool sections:**
  - The IMPACT tool includes seven main sections:
    - Instructions - brief guidance on how to use the tool
    - Patient demographics – to ensure accurate patient identification
    - Risk categories (red, amber, and green) and lists of risk indicators below each category – outlines the risk factors that contribute to the patient’s risk classification
    - Supplementary boxes for high-risk medicines and physical health issues – provide subcategories of these risk indicators
    - Outcome section – outlines the final risk classification for the patient
    - Comment section - staff can use this section to add any relevant notes
    - Staff details – for documentation and traceability
- **Instructions on using the IMPACT tool:**
  1. Pharmacy staff should start by reading the instructions and completing patient details.
  2. Go through the list of risk indicators ticking the boxes that apply to the patient. If some risk indicators are not applicable, options may include either leaving them blank or writing ‘NA’.
  3. Use the comment section, if needed, to add more details about the patient, specify required actions, or any other need.
  4. Once all the risk indicators are reviewed, including the supplementary boxes if required, pharmacy staff should select the appropriate risk category in the outcome section. If they disagree with the outcome for any reason, they should use their clinical judgement by ticking the ‘other’ tick box under the risk group they believe the patient should be classified into, and writing the reason in the free text space.
  5. Complete the staff details at the end of the tool and record the date of assessment.
  6. Lastly, ensure appropriate documentation.
- **Important considerations:**
- Make sure to count the number of amber risk indicators applicable to the individual patient following scoring. If the patient is affected by four or more amber risk indicators, go back to the red category and tick ‘Patient has > 4 amber criteria’.
- If you want to record more details about certain risk indicators, use the comment box. For example, if you tick ‘patients with physical healthcare issues requiring follow-up by pharmacy team’, you can describe the issues in the comment box such as: ‘patient has an acute infection and requires review of antibiotics’ or ‘patient has increased prolactin’ or ‘patient has uncontrolled diabetes/ uncontrolled blood pressure’. Another example could be with the risk indicator ‘Patients planned for discharge/leave with outstanding issues requiring follow up’. The outstanding issues could be described in the comment section.
- Note that information about some risk indicators may not always be available (e.g. blood test results). These could be skipped and should be completed once available.
- Whilst the tool includes many modifiable risk indicators that pharmacy team members may try to address, it is important to understand that not all risk factors are modifiable (e.g. age). Additionally, some risk indicators may not change quickly for some individuals (e.g. self-harm and suicidal thoughts) or may not result in any direct change in care (e.g patients lacking capacity to consent to medication administration). These risk indicators may not be directly linked with negative consequences but are there to highlight that these patients may require more attention. This means that patients with these risk indicators may always be amber, which is OK. However, if pharmacy team members believe that a patient should be de-escalated to green, they can use their clinical judgement through the ‘other’ tick box to de-escalate the patient and note down the reason.
- Many of the risk indicators are open to interpretation and this was deemed necessary to ensure the flexibility of the tool. Clinical judgement is important and should always be utilised alongside the tool. For example, there is no timeframe for the risk indicator ‘patient missed > 2 doses of the same regular prescribed medication’. Clinical judgement should be used here as the timeframe will be different based on the type of medication missed (e.g. depot antipsychotics, antibiotics, etc.). Additionally, for the risk indicator “Increase of a regular psychotropic within 7 days of the last increase (unless as part of a dose titration regimen)”, pharmacy team member completing the tool may consider a titration regimen to be an amber and could escalate the patient.
- The tool does not alert pharmacy staff when there are changes in the patient situation and it does not capture every eventuality. It is important to use the tool alongside existing systems to be alert to changes in patient circumstances. For example, you might learn during the ward round that a patient previously rated as ‘green’ using the IMPACT tool may be started on clozapine. You then need to review the tool and escalate the patient. Another example could be a newly admitted patient, categorised as ‘green’ by the tool as they do not have any prescribed medications. You might learn from a colleague that they need some advice on antipsychotic treatment choice. You might then review the tool and escalate the patient, perhaps using the ‘other’ tick box.

# **5. Implementation of the IMPACT tool**

- The IMPACT tool should be introduced to staff by explaining the benefits of using a prioritisation tool, previous experiences, and how the tool was developed.
- At least one training session should be delivered to pharmacy team members prior to implementing the IMPACT tool (refer to the training section below).
- Please consider the following factors that may contribute to successful implementation of patient prioritisation tools (these were identified through interviews with UK pharmacists who used tools or processes to prioritise their mental health inpatients):
  1. **Support:**
     - Having strong support from the leaders.
     - Having strong support from the multidisciplinary team.
     - Having a source of advice (e.g. medicines safety group)
  2. **Team:**
     - Championship and engagement from the team.
     - Involving the team in decisions regarding implementation of the tool.
  3. **Education:**
     - Setting realistic expectations.
       - During the initial implementation of this tool, pharmacy staff may encounter challenges, including difficulties with its use and maintaining the recommended review frequency. It is essential to proactively identify and address these issues. Open communication and collaborative problem-solving will be crucial in ensuring staff feel supported.
     - Introducing the tool as an assistant not instructor.
       - Emphasising the tool's role as a supportive resource will foster positive adoption
     - Consistency and follow up through meetings.
       - Consistent team discussions are crucial for understanding the nuances of tool usage, enabling appropriate adaptation and ensuring the tool aligns seamlessly with the team's needs and daily workflow.

# **6. Documentation of IMPACT tool use**

- Regardless of the format used (printed or Excel), ensure that the completed checklist is properly documented in the patient's medical record. Follow your organisation's policies and procedures for documentation.

# **7. Feedback and Improvement**

- We encourage regular discussions and feedback with the team to ensure appropriate adaptation to your specific context.

# **8. Training**

Appropriate training is key in using the tool effectively. Training materials are provided in **Appendix 1** and should be adapted and updated based on your organisation’s context. Ensuring staff can readily access risk indicator information is essential for effective tool utilisation. For instance, when assessing ‘self-harm or suicidal thoughts’ staff require clear guidance on where this data is normally recorded. If such documentation is absent, staff should be guided to either document the finding or apply their clinical judgment. Furthermore, consider reviewing current documentation practices to identify potential modifications that could facilitate easier tool completion.

Experts who participated in the tool development agreed that:

- At least one training session should be delivered to pharmacy team members prior implementing the IMPACT tool.
- Training in how to use the IMPACT tool should be available for staff new to mental health services.
- Training should include the benefits of using the tool for staff whether they are new or experienced in mental health inpatient care.
- Training should include the importance of balancing standardisation and clinical judgement when prioritising patients for care.
- Training should include some worked examples for the tool (see **Appendix 2**).
- Training should include some feedback from previous users of the tool (to be added locally).

# **9. Adaptation of the IMPACT tool for Different Contexts**

- **Introduction:**
  - This tool was designed to be adaptable to various clinical settings. The following guidelines will help you tailor the tool to your specific context.
- **Frequency of review:**
  - The frequency of review in the tool is for guidance. Each organisation should adapt the frequency based on their local context.
  - The initial implementation phase of the IMPACT tool in your organisation should help in determining the appropriate frequency of review. Make sure to consider the variation across different wards and hospitals, and involve all pharmacy team members in evaluation and determination of the appropriate frequency of review.
  - Note that some pharmacy staff may have the capacity to review all their local inpatients daily and question the benefit of the tool. It is important to have a discussion with them to emphasise the other benefits of using the tool such as facilitating handover, benchmarking, accountability, and helping with staffing allocation. In addition, note that research from acute care suggests spending more time reviewing high-risk patients may be more beneficial than reviewing all patients rapidly (Falconer et al., 2019). In addition, other acute care pharmacy studies from the UK and Ireland reported that deprioritising patients categorised as ‘lower risk’ save time and resources (Clarke et al., 2023 & Geeson et al., 2019)
- **Different Specialties (e.g., psychiatric intensive wards, older people):**
  - Some risk indicators may not be relevant in certain wards. For example, some pharmacy staff may find that the majority of their patients in an older adult ward are older than 70 years. This may result in categorising most of their patients as high-risk patients when according to their clinical judgement they are not. Consequently, they may consider removing the risk indicator ‘age>70’ following thorough consideration and team discussions.
  - Caution should be taken when removing risk indicators as these were identified through rigorous research and their continued presence may be safer than their elimination. For example, older people or children may be admitted to acute adult wards in certain situations. Removing the age risk indicators in these wards may risk overlooking these patients.
- **Reference ranges and medications lists:**
  - Reference ranges for parameters are expected to be locally added to the tool.
  - While examples are included in the tool for QT prolonging medications and medications requiring intensive therapeutic drug monitoring, these are only for guidance. Organisations may have agreed lists of these medications that could be used alongside the tool, yet care must be taken as including all medications, such as all antipsychotics, may risk overclassifying patients as medium-risk when they may be low-risk.
- **Partially completing the tool:**
  - Considering the length of the tool, it was previously suggested that pharmacy team members may stop completing the tool once a red indicator is ticked. This was believed to speed up the prioritisation process as the patient will be directly categorised in the red category. Caution should be taken when such approach is used as there was no agreement on using this approach by experts involved in the tool development. Following this approach may risk missing information about the patients and may affect the handover process.
- **Manual and training material:**
  - The ‘frequently asked questions’ section of this manual can be adapted by modifying or adding new questions that may arise during the training sessions or team meetings.
  - The training material can also be adapted by adding more explanations or adding more specific or relevant examples. Importantly, pharmacy technicians and pharmacy staff new to mental health may require more specific training. For example, some of the terminology (e.g. T2 and T3 forms), risk indicators (e.g. creatine kinase), and interpretation of blood tests reference ranges could be explained. Clarifying what is meant by high-risk medicines is also important, e.g. whether the indication is to be considered. Additionally, feedback from previous users of the tool should be sought and included in the training.
  - It is encouraged to continuously update the ‘frequently asked questions’ and the training sessions when questions or issues arise in the team meetings to ensure improved training for future staff.

# **10. Support mechanisms**

This section is to be completed locally. It should outline how team members can reach out for support and from whom they can seek this. It should be continuously reviewed and updated to ensure that pharmacy team members are aware of how to get support.

# **11. Frequently asked questions**

- - **Q:** What do I do when I disagree with the tool outcome?
  - **A:** You can use your clinical judgement through the ‘other’ tick box to escalate or de-escalate a patient. Make sure to include the reason in the space next to the word ‘other’.
  - **Q:** What do I do if I am uncertain of some risk indicators or the final risk category for the patient?
  - **A:** You can use the comment box at the end of the tool to add notes so that other team members can understand your concerns when they review the tool. If you require direct support, reach out to your line manager or follow the appropriate support mechanism as described in section 10.
  - **Q:** Can I modify the tool?
  - **A:** As described in section 9 of this document, adaptation of the tool may include some modification. However, this should be approached with caution considering the implications of the changes with patient safety as the top priority.

**12. References**

- Alshaikhmubarak F.Q., Keers R.N. & Lewis P.J. Potential Risk Factors of Drug-Related Problems in Hospital-Based Mental Health Units: A Systematic Review. *Drug Saf* **46**, 19–37 (2023). https://doi.org/10.1007/s40264-022-01249-1
- Clarke R., Colleran M., Melanophy G. & Bermingham M. Enhancing the clinical pharmacy service of a large teaching hospital: Development of a new clinical prioritisation tool. *Exploratory Research in Clinical and Social Pharmacy* **12**, (2023). https://doi.org/10.1016/j.rcsop.2023.100335.
- Falconer N., Barras M., Cottrell N. How hospital pharmacists prioritise patients at high-risk for medication harm. *Res Social Adm Pharm* **15** (10): 1266-1273 (2019). https://doi.org/10.1016/j.sapharm.2018.11.003
- Geeson C., Wei L., Franklin BD. Development and performance evaluation of the Medicines Optimisation Assessment Tool (MOAT): a prognostic model to target hospital pharmacists' input to prevent medication-related problems. *BMJ Qual Saf* **28** (8):645-656 (2019). https://doi.org/10.1136/bmjqs-2018-008335

**
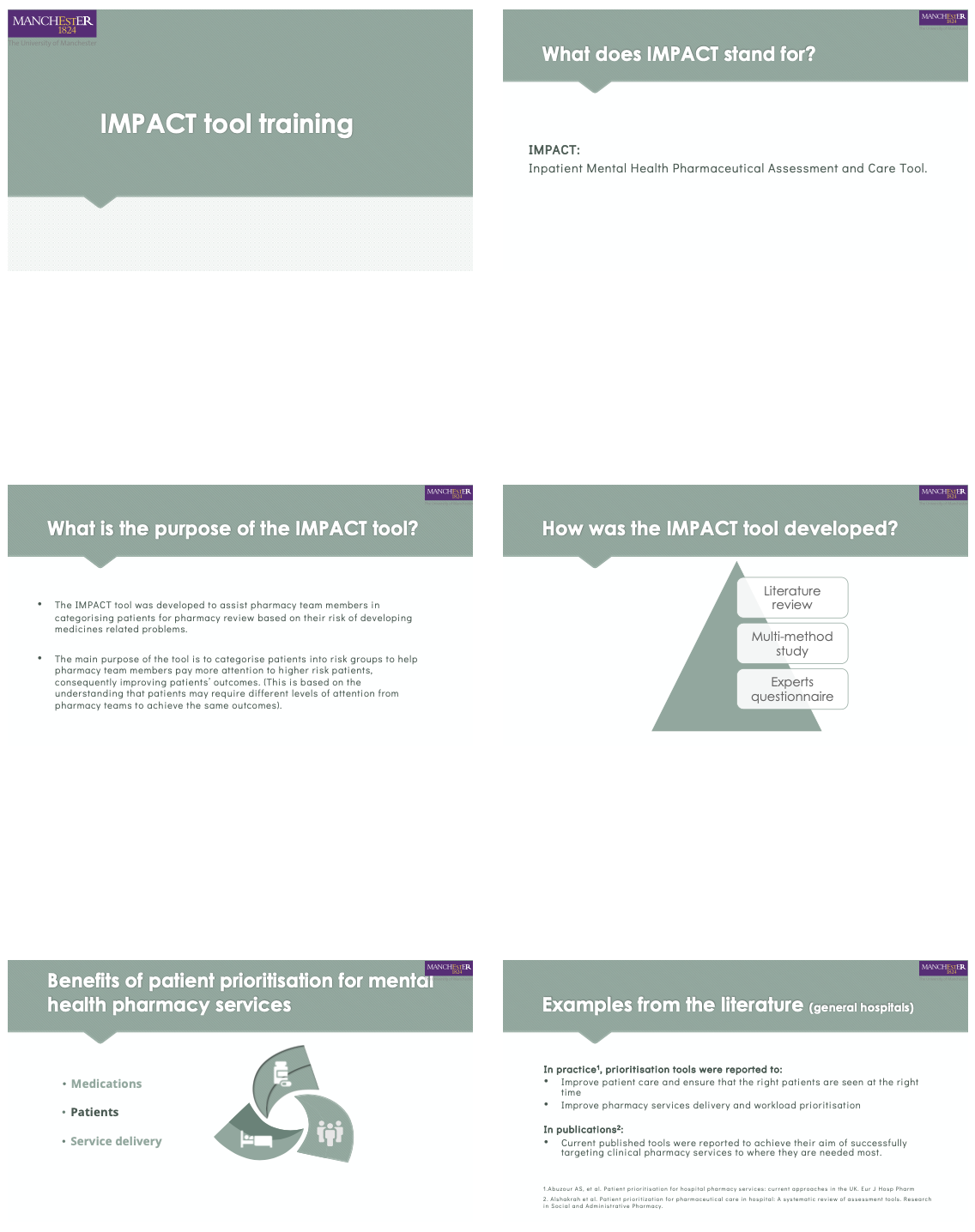
Appendix 1**

**
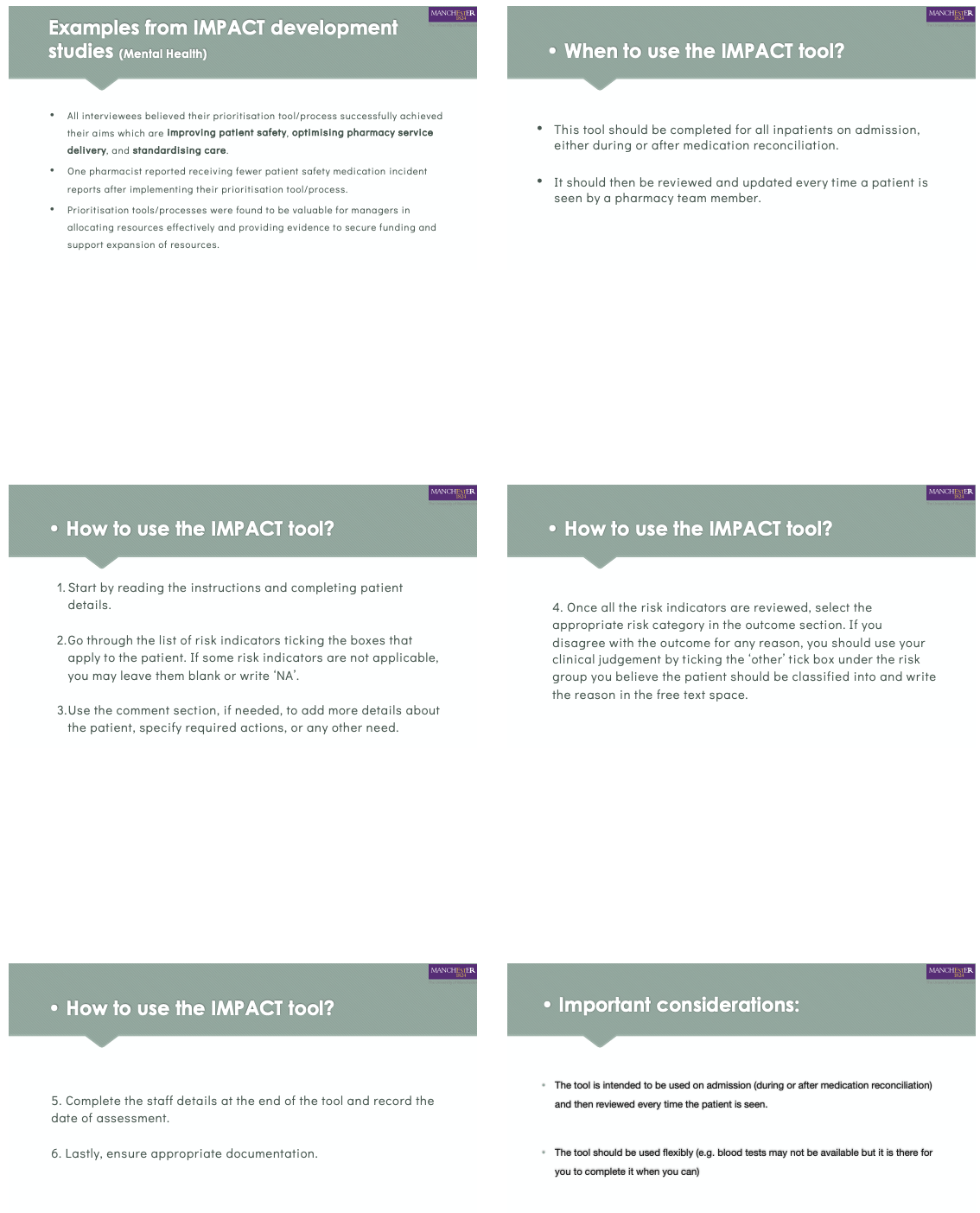
**

**
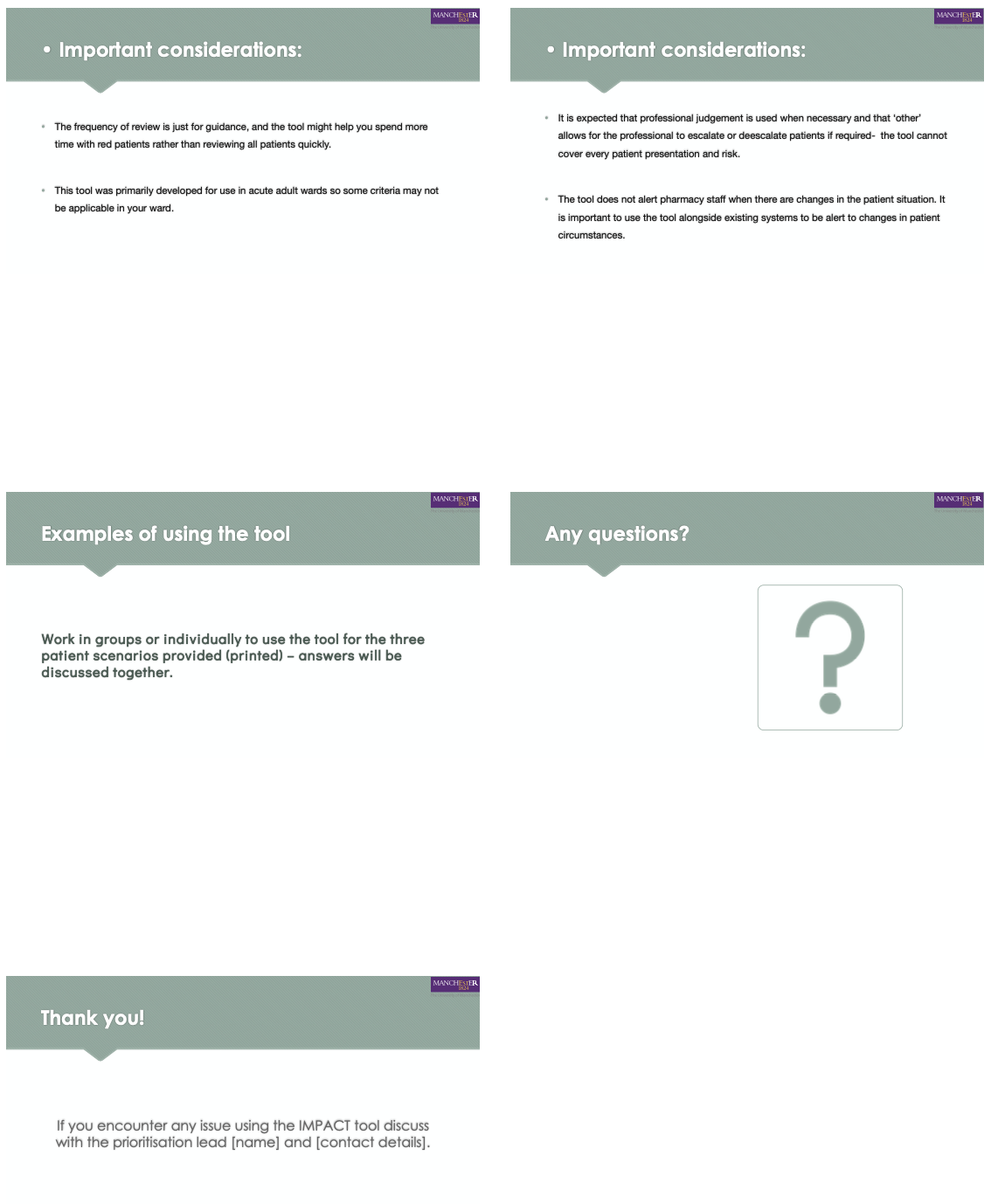
**

**Appendix 2**

**Examples for using the IMPACT tool**

Below are five examples illustrating potential application of the IMPACT tool. These are intended as illustrative guides only. Real-world cases are complex and require the application of professional clinical judgment.

**Example one**

Name: Mr. John Smith Age: 32 years Diagnosis: Schizophrenia

**Background:** John has a 10-year history of schizophrenia and has had several previous admissions to acute mental health wards. He is now admitted due to increased agitation, auditory hallucinations, and paranoia. Historically, John has poor adherence with his antipsychotic medication.

**Current Medication (**after medication reconciliation)

- Risperidone 3 mg twice daily (oral)
- Lorazepam 1 mg PRN for agitation (oral, up to 3 times a day)
- Zopiclone 7.5 mg at night for sleep

**Situation**

John’s recent relapse was triggered by non-adherence to his oral antipsychotic treatment, citing concerns about side effects and not feeling the need for medication. On admission, he was extremely agitated, had difficulty sleeping, and was experiencing vivid auditory hallucinations. His physical health was stable, and he has no known allergies. John has a history of smoking and occasionally uses cannabis, which he states helps him calm down.

**Pharmacy Review**

Medication Adherence: The pharmacy team is consulted to assess John’s adherence issues. The team conducts a medication review and identifies potential side effects contributing to non-adherence (sedation and weight gain from risperidone). The team considers switching to an alternative depot antipsychotic because of adherence issues.

**Prioritisation tool outcome:** Amber

1. More than one anxiolytic/hypnotic (one is prn but may be taken regularly as patient is agitated)
2. Non adherence (it was due to side effects, so may change after treatment is changed)

* Might escalate to Red if IM rapid tranquillisation is administered.

**Example two**

Name: Mrs. Sarah Williams Age: 78 years

Diagnosis: Alzheimer’s Disease with Behavioral and Psychological Symptoms of Dementia (BPSD)

Background: Sarah is newly admitted to the older adult mental health ward due to increasing aggression, wandering, and periods of confusion at home. She lives with her daughter, who is struggling to cope with the severity of her symptoms. Sarah has a history of hypertension and osteoarthritis. Her dementia was diagnosed three years ago and has gradually worsened. She has no known allergies.

**Current Medication (**after medication reconciliation)

- Donepezil 10 mg once daily (oral)
- Amlodipine 5 mg once daily (oral) for hypertension
- Paracetamol 1 g three times daily for osteoarthritis pain
- Oxybutynin 5 mg twice daily (oral)

**Situation**

During her admission, Sarah becomes increasingly agitated, displaying aggression towards staff and other patients. She is experiencing visual hallucinations and nighttime restlessness. Her sleep pattern is highly disrupted, and her agitation worsens in the evening, leading to what her daughter describes as “sundowning.”

**Pharmacy Review**

Antipsychotic Use: The medical team considers using Risperidone (0.5 mg daily) to manage Sarah’s aggressive behavior. The pharmacy team is asked to review the appropriateness of antipsychotic use in older adults with dementia, given the increased risk of cerebrovascular events and mortality.

**Prioritisation tool outcome: Red**

Patient with dementia prescribed one or more antimuscarinics

**Amber criteria**

1. Age >70

**Example three**

**Patient Details**

**Name**: Ms. Rachel Johnson **Age**: 28 years

**Diagnosis**: Bipolar Disorder Type I, current manic episode

**Background**: Rachel has a history of bipolar disorder with multiple hospital admissions for manic and depressive episodes. She was admitted following a recent manic episode, characterised by increased energy, rapid speech, delusional beliefs that she is aristocracy, and impulsive spending. Rachel is newly admitted and reports that before her admission she stopped her mood stabiliser because she “feels great” and didn’t see a need for medication.

**Current Prescribed Medication (**after medication reconciliation)

- **Lithium** 25 mg once daily (oral)
- **Olanzapine** 10 mg once daily (oral) (initiated during admission to address the mania)
- **Lorazepam** 1 mg PRN (up to three times daily for acute anxiety/agitation)

**Situation**

Rachel is highly agitated, not sleeping, and expresses grandiose ideas. She refuses her lithium medication, citing concerns about side effects like weight gain and tremors. Her physical health is stable. There is limited information available from the patient regarding allergy due to mental state, and GP practice is closed (weekend).

Prioritisation tool outcome: Red

1. High risk medicine - Lithium

Amber criteria:

1. Patient administered oral ‘when required’ psychotropic for agitation.

2. Non adherence (it was due to side effects, so may change after treatment is changed).

3. Undetermined allergy status.

**Example four**

**Patient Details**

**Name**: Mr. Edward Turner

**Age**: 82 years

**Diagnosis**: Major Depressive Disorder with psychotic features

**Background**: Edward has a long-standing history of depression but was admitted last week for worsening depressive symptoms, including psychotic features (hearing voices). He has no previous history of psychosis. His medical history includes **Type 2 Diabetes** and **Hypertension**. Edward lives alone, and his family noticed that he had stopped taking care of himself and wasn’t eating properly. He also missed doses of his diabetes and blood pressure medications.

**Current Medication**

- **Sertraline** 100 mg once daily (oral)
- **Aripiprazole** 5 mg daily (oral) for psychotic features
- **Metformin** 1g MR once daily (oral) for Type 2 Diabetes
- **Ramipril** 5 mg once daily (oral) for Hypertension
- **Amlodipine** 10mg once daily (oral) for Hypertension
- Atorvastatin 20mg once daily (oral) for cardiovascular disease prevention

**Situation**

Edward reports feeling hopeless and hearing voices that tell him he is worthless. His appetite is poor, and he is sleeping most of the day. His blood sugar control has worsened, and his family expresses concern about his declining health. His physical exam reveals weight loss and low energy. He has no known allergies.

Part.1:

**Prioritisation tool outcome:** **Red**

Patient have > 4 amber criteria.

1. Age > 70

2. Non adherence

3. Patients with physical healthcare issues requiring follow-up by pharmacy team

4. Polypharmacy > 5 regular medications

**Part 2**

**While reviewing Edward’s situation today, his blood sugar was controlled and his blood pressure is within the normal range.**

**Prioritisation tool outcome:** Amber

Amber criteria:

1. Age > 70

2. Non adherence

3. Polypharmacy > 5 regular medications

**Example five**

**Patient Details**

- **Name**: Mr. Raju Khan
- **Age**: 40 years
- **Diagnosis**: Generalized Anxiety Disorder (GAD) and Alcohol Use Disorder
- **Background**: Raju was admitted after presenting with severe anxiety, tremors, and confusion. He has a history of GAD and alcohol use disorder. His alcohol intake has increased in recent months, leading to a state of **alcohol withdrawal** upon admission. Raju is also experiencing **insomnia** and is constantly on edge, with panic attacks occurring multiple times a day. He has no known allergies.

**Current Medication**

- **Chlordiazepoxide** reducing regime plus PRN for alcohol withdrawal
- **Propranolol** 40 mg twice daily for anxiety
- **Fluoxetine** 40 mg daily for GAD
- **Pabrinex** 1 pair once daily intramuscularly to prevent Wernicke's encephalopathy (5 days) (please ignore current supply issues as part of this case)

**Situation**

Raju has been consuming alcohol daily and reports that it helps him cope with his anxiety. On admission, he was shaking and sweating profusely, with a fast heart rate. His blood alcohol level was high, and he was placed on a **benzodiazepine withdrawal regimen**. He also reports difficulty sleeping, which exacerbates his anxiety. His physical health is mostly stable, but there are concerns about **liver function** due to his alcohol use.

**Prioritisation tool outcome:** Amber

1. Alcohol detox medications (Pabrinex and/or Chlordiazepoxide)

2. Patients with substance abuse
